# Editorial: Lifetime achievements in avian physiology

**DOI:** 10.3389/fphys.2026.1816782

**Published:** 2026-04-01

**Authors:** Colin G. Scanes, Sandra G. Velleman

**Affiliations:** 1 Department of Biological Science, University of Wisconsin Milwaukee, Milwaukee, WI, United States; 2 Department of Animal Sciences, The Ohio State University, Wooster, OH, United States

**Keywords:** growth hormone, intestinal development, met-enkephalin, mitochondria, neuroendocrine, satellite cells, sexual dimorphism, stress

Lifetime Achievements in Avian Physiology topic provides a tantalizing account of achievements and philosophy of a series of senior avian physiologists. These papers represent only a fraction of the giants in research in avian physiology, specifically the physiology of chickens and other poultry species. The senior researchers are considered in alphabetical order of their last names.

Walter Bottje has had an illustrative research career. He states his passion as “physiology was really what floated my boat”. His research career in avian physiology began with a series of studies on heat stress in broiler chickens. Among his achievements include the following:

- Relationships between celiac blood flow and prostaglandins
- Oxidative stress/glutathione (GSH)/Reactive oxygen species (ROS)
- The functioning a of mitochondria in muscle and how this affects feed efficiency
- Mitochondrial dysfunction including defects in the electron transport mechanism

and what he describes as “the gene and gene product landscape associated with feed efficiency”.

Erf has had a distinguished research career in basic and applied avian physiology. Among her research achievements in poultry included the following:

- The role of maternally derived immunoglobulins
- Relationships between genetics and stressors on the immune system
- Improving poultry health by immunomodulators.

Wayne Kuezel spent sabbaticals at the Roslin Institute (Scotland), Justus Liebig University Giessen (Germany) and Nanjing Agricultural University (China). During these, he gained from immersive collaboration catalyzing his overall research career going from strength to strength. Among the stellar contributions of Wayne Kuezel are the following:

- The development of a stereotaxis atlas for the chicken’s brain.
- The distribution of gonadotropin releasing hormone (GnRH) containing neurons and fibers in the avian brain.
- Advances in our understanding the neuroendocrine basis of stresses such as feed deprivation and restraint in chickens.

Krystyna Pierzchała-Koziec has had an outstanding research career. Her achievements have been in four, somewhat overlapping, areas:

- Stress physiology in chickens and livestock
- The physiology of Met-enkephalin
- Hormonal and neuroendocrine control of metabolism
- Fostering world-class basic and applied research in Poland and throughout Europe.

Scanes was drawn to avian physiology by two wonderful mentors: the late Professor John Phillips and Professor Sir Brian Follett in the UK. His research has included a prolonged focus on growth hormone (GH). His paper discusses GH effects in chickens on growth, lipid metabolism, reproduction, neural development and angiogenesis together with the control of GH release. He also introduces a series of questions that have not been addressed.

Production of chicken meat has expanded greatly over the last 50 years. According to FAOSTAT, production was 7.56 Mt (megatonne) in 1961, increasing to 15.9 Mt in 1974, 56.9 Mt in 1999 and 123.8 Mt in 2024. These dramatic increases can be attributed to genetics (growth being moderately heritable), nutrition, poultry health and management. We were delighted that Siegel agreed to contribute to this topic. He is the “father of poultry genetics”. He chose to focus on one aspect of his long and on-going research career, namely; sexual dimorphism in body weight in chickens. When this is considered on a proportional manner, there is continuing sexual dimorphism in chickens selected for growth (high body weight and low at eight weeks old) over 67 generations. This work and the physiology of sexual dimorphism in birds is also discussed in a commentary by Scanes.

Uni has an outstanding record of achievement in avian physiology. Her research can be classified as comprehensive and impactful.

- Development of the gastro-intestinal tract of chickens
- *In ovo* feeding in chickens and how *in ovo* feeding influences the proliferation, differentiation and maturation of epithelial cells in the small intestine
- The molecular basis of taste.

In 1955, U.S. Air Force Major General Frederick C. Blesse stated “No Guts, No glory”. This adage became one of Zehava Uni’s principles for a successful researcher. She also included the importance of resilience to adversity, flexibility and following “your true passion”.

Sandra Velleman’s interest in science was fostered from a very young age by her mother. One teacher recognized her research skills at a time of life when she was not even sure what a research scientist was. We are fortunate that she went on to an exemplary research career. Throughout her career, Sandy Velleman’s guiding predisposition was that extracellular matrix is a critical important component in non-connective tissues including muscle despite the then paradigm that extracellular matrix was not. She took the “risk” to challenge the prevailing paradigm. Her laboratory was the first to demonstrated that satellite cells produce extracellular matrix proteoglycans using chicken breast muscle satellite cells. Velleman has a series of other land-mark achievements, namely, the following: 1. satellite cells are heterogeneous; 2. muscle development of muscle and consequently meat is influenced by extrinsic conditions (temperature and nutrition) and 3. maternal inheritance of aspects of muscle morphology.

There are some common themes from the papers and the distinguished avian physiologists. These include the following:

- The importance of mentors, collaborators and others providing the stimulus for research direction and the development of research programs.
- The importance of passion and willingness to challenge prevailing paradigms and/or develop a focuses research program(s).
- Collaborators and collaboration play a pivot role in research in avian physiology.

A common theme is the role of mentors. Examples of the sway of mentors include the late Paul Harrison (University of Illinois) for Walter Bottje and both Robert Etches and Robert Marsh for Gisela Erf. Turning to collaboration, the partnership between Sandy Velleman and Douglas McFarland was very fruitful building on the strengths of both researchers. Another example of close, successful and ongoing collaboration is that between Pierzchała-Koziec and Scanes on stress and opioid peptides. Bottje highlights the effect of a single discussion with Robert Wideman. Research lives have been changed by people and environments. It is interesting to note that three of the contributions come from the University of Arkansas, Center of Excellence in Poultry Science.

There are two quotations that provide insight into to the role of luck in research “Give me lucky generals” and what is a pertinent rejoinder “I am a great believer in luck, and I find the harder I work, the more I have of it”; these being attributed, albeit falsely or at least without supporting evidence, to respectively to Napoleon Bonaparte (1769-1821) and Thomas Jefferson (1743 - 1826). We are left with the statement from Louis Pasteur stated that “In the fields of observation chance favors only the prepared mind” in 1854 (Leonard, 2013).
